# Rare Copy Number Variants Identify Novel Genes in Sporadic Total Anomalous Pulmonary Vein Connection

**DOI:** 10.3389/fgene.2018.00559

**Published:** 2018-11-23

**Authors:** Xin Shi, Liangping Cheng, XianTing Jiao, Bo Chen, Zixiong Li, Yulai Liang, Wei Liu, Jing Wang, Gang Liu, Yuejuan Xu, Jing Sun, Qihua Fu, Yanan Lu, Sun Chen

**Affiliations:** ^1^Department of Pediatric Cardiovascular, Xinhua Hospital, School of Medicine, Shanghai Jiao Tong University, Shanghai, China; ^2^Department of Medical Oncology, Bayi Hospital, Nanjing University of Chinese Medicine, Nanjing, China; ^3^Institute of Health Sciences, Shanghai Institutes for Biological Sciences, Chinese Academy of Sciences, Shanghai, China; ^4^Department of Cardiothoracic Surgery, Xinhua Hospital, School of Medicine, Shanghai Jiao Tong University, Shanghai, China; ^5^Medical Laboratory, Shanghai Children’s Medical Center, School of Medicine, Shanghai Jiao Tong University, Shanghai, China

**Keywords:** congenital heart defects, total anomalous pulmonary venous connection, whole-exome sequencing, copy number variants, pathogenesis

## Abstract

Total anomalous pulmonary venous connection (TAPVC) is a rare congenital heart anomaly. Several genes have been associated TAPVC but the mechanisms remain elusive. To search novel CNVs and candidate genes, we screened a cohort of 78 TAPVC cases and 100 healthy controls for rare copy number variants (CNVs) using whole exome sequencing (WES). Then we identified pathogenic CNVs by statistical comparisons between case and control groups. After that, we identified altogether eight pathogenic CNVs of seven candidate genes (*PCSK7, RRP7A, SERHL, TARP, TTN, SERHL2, and NBPF3)*. All these seven genes have not been described previously to be related to TAPVC. After network analysis of these candidate genes and 27 known pathogenic genes derived from the literature and publicly database, *PCSK7* and *TTN* were the most important genes for TAPVC than other genes. Our study provides novel candidate genes potentially related to this rare congenital birth defect (CHD) which should be further fundamentally researched and discloses the possible molecular pathogenesis of TAPVC.

## Introduction

Total anomalous pulmonary venous connection (TAPVC) is a rare but heterogeneous congenital heart anomaly in which pulmonary veins do not connect routinely to the left atrium but abnormally connect to the right atrium or systemic venous system. The incidence of TAPVC is approximately 1 out of 15,000 live births (Ammash et al., 1997; Bjornard et al., 2013; Thummar et al., 2014). TAPVC is rare but without proper intervention in the first year of life the mortality of TAPVC is nearly 80% (Burroughs and Edwards, 1960). However, the molecular mechanism of TAPVC remains unknown.

So far, only a few genes have been demonstrated as pathogenic genes for TAPVC and these genes are just a partial explanation for some patients. Bleyl et al. (2006) used genetic linkage analysis found a locus for TAPVR at 4p13-q12 called total anomalous pulmonary venous return 1 (*TAPVR1*) and other important pathogenic genes in this region include vascular endothelial growth factorreceptor 2 (*VEGFR2*) and platelet-derived growth factor receptor α (*PDGFRA*). Nash et al. (2015) used whole genome sequence to identify a non-synonymous variant in the retinol binding protein 5 (*RBP5*) gene which probably related to TAPVC. Li et al. (2017) considered activin A receptor type II-like 1 *(ACVRL1*) and sarcoglycan delta (*SGCD*) as TAPVC pathogenic genes using whole exome sequence from 6 TAPVC cases. However, these pathogenic genes explain only a small fraction of the molecular mechanism of TAPVC, the underlying cause in most patients remains unknown.

Copy number variant (CNV) is defined as a segment of DNA at least 1 kb in size that differs in copy number compared with a representative reference genome (Wellcome Trust Case Control Consortium et al., 2010; Pinto et al., 2011). CNVs have been shown to play an important role in the pathogenicity of complex birth defects (Greenway et al., 2009). CNV, or submicroscopic chromosomal deletions or duplications, has emerged as an important contributor to congenital genetic disorders and has identified critical dosage sensitive genes important for cardiac development (Hitz et al., 2012; Southard et al., 2012; Mlynarski et al., 2015). Whether CNV detection could be as a genetic selection for novel pathogenesis genes of TAPVC is still not reported previously, and it needs to be further studied.

## Materials and Methods

### Patient Ascertainment

Our study recruited patients with TAPVC Xinhua Hospital Affiliated to Shanghai Jiao Tong University School of Medicine whose diagnoses were confirmed by echocardiography, cardiac catheterization, computed tomography, and other medical recordings. Patients with multiple major developmental anomalies, developmental syndromes, or major cytogenetic abnormalities were excluded. Ethical approval was given by the medical ethics committee of Xinhua Hospital.

### Detection of CNVs From WES Data

Peripheral blood samples were obtained and DNA was extracted using the QIAamp DNA Blood Midi Kit (Qiagen, Germany). WES samples were captured with the Agilent Sure Select Target Enrichment kit (V6 58 Mb; Agilent Technologies, United States) and sequenced on the Illumina HiSeq 2500 platform (Glessner et al., 2014; Li et al., 2015). CNV coordinates were converted to the GRCh37/hg19 build using the UCSC Genome Browser LiftOver tool. CNVs with 50% or larger overlap with telomere, centromere, segmental duplications, or immunoglobulin regions were excluded (Hanemaaijer et al., 2012). After filtering we screened out rare CNVs by comparing with the Database of Genomic Variants (DGV^1^) and Online Mendelian Inheritance in Man (OMIM^2^).

### Identification of Pathogenic CNV Candidates

The CNV regions were firstly annotated with RefSeq genes. For each gene and each sample, the copy number status was determined by the annotated CNV regions. The pathogenic CNV candidates were then identified by statistical comparisons between the case and control groups. The statistical comparisons between groups were analyzed by one-side Fisher’s exact test with alternative hypothesis that the mutation frequency is greater in case group than the control group. The CNV candidates were defined as potentially pathogenic if the *P* < 0.01. The analysis and visualization were implemented in R programming software with version 3.5.0.

### Protein–Protein Interaction (PPI) Analysis

Protein–Protein Interactions (PPI) are physical contacts with molecular associations between chains that occur in a cell or in a living organism in a specific biomolecular context (De Las Rivas and Fontanillo, 2010). Our candidate pathogenic genes with CNVs, combined with 27 known disease-causing genes derived from the literature and publicly available database, were mapped to PPI network in STRING database^3^ (Brohee et al., 2008), which identified the connections between the candidate pathogenic genes and the known disease-causing genes. Information found in STRING databases supports the construction of interaction networks (McDowall et al., 2009).

### Expression Patterns of the Selected Genes During Human Embryonic Heart Development

Expression patterns of the human embryonic heart of candidate genes were detected using an Affymetrix HTA 2.0 microarray. To determine whether these candidate genes could affect human embryonic heart development, Carnegie stages 11 through 15 of human embryonic heart samples were collected from Xinhua hospital. RNA extraction used TissueLyser II (Qiagen, Germany) and the RNeasy MinElute Cleanup Kit (Qiagen, Germany) as previous study (Nolan et al., 2006). The integrity and purity of the RNA was detected by the Experion automated gel electrophoresis system (Bio-Rad, United States) and the NanoDrop 2000c spectrophotometer (Thermo Fisher Scientific, United States).

## Results

### Clinical Data

A total of 78 sporadic TAPVC cases and 100 healthy controls were recruited in our research. Among these patients, no one had central nervous system malformations, vertebral defects, or genitourinary malformations. The patients’ ages ranged from 27 days to 7 years; 45 patients were male (57.7%) and 33 were female (42.3%). Among all these patients, 47 had atrial septal defect (ASD), 16 had patent foramen ovale (PFO), 10 had ventricular septal defect (VSD), and 16 had patent ductus arteriosus (PDA). Double outlet right ventricle (DORV) was discovered in three patients, atrioventricular septal defect in three patients. The detailed clinical data and cardiac phenotypes are summarized in Table 1. All patients were recruited via Xinhua Hospital Affiliated to Shanghai Jiao Tong University School of Medicine and all signed an informed consent approved by the Ethics Committee of Xinhua Hospital.

**Table 1:** The summary of 78 TAPVC patients.

Patient characteristics	Discovery cohort
Mean age at diagnosis (years)	0.95 ± 1.87
BMI (kg/m^2^)	14.92 ± 3.05
Male (*n*, %)	45(57.7)
Mortality (*n*, %)	4(5.1)
**Associated cardiac lesion (*n*, %)**	
ASD	57(73.7)
PFO	16(20.5)
VSD	10(12.8)
PDA	16(20.5)
DORV	3(3.8)
AVSD	3(3.8)

### CNVs in Patients With TAPVC and Identification of Candidate Genes

**FIGURE 1:**
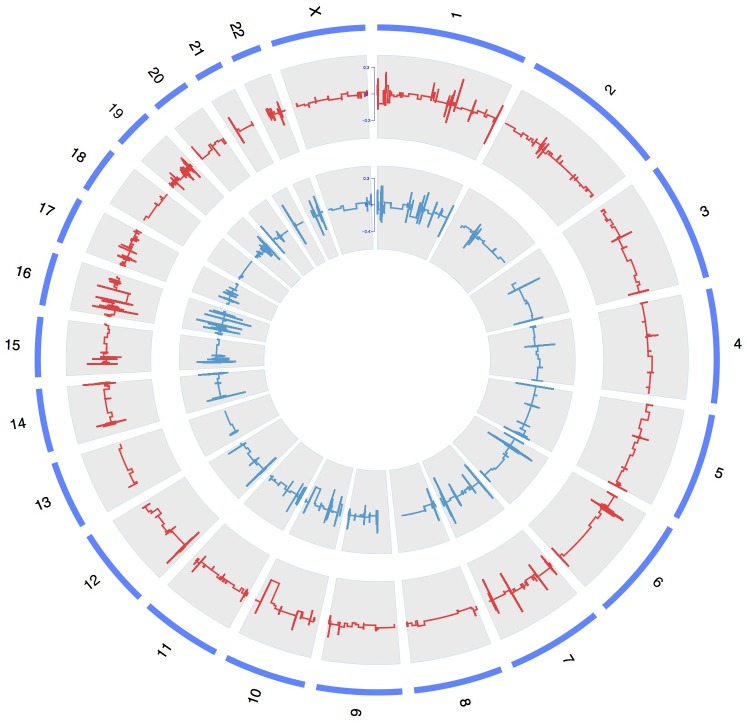
A whole-genome view of copy number variations in case and control groups. Circos plot for variants visualization with broad horizontal area from chromosome level. The outer, middle, and inner tracks display the chromosomes, CNV frequency in case group, and CNV frequency in control group. The lines above or under zero represent gain or loss.

To discover the pathogenic CNV candidates, we identified WES data by statistical comparisons between the case and control groups. We use circos plot for CNV visualization with broad horizontal area from chromosome level (Figure 1). In all chromosomes, chromosome 1 had the most CNV numbers than other in our patients.

**FIGURE 2:**
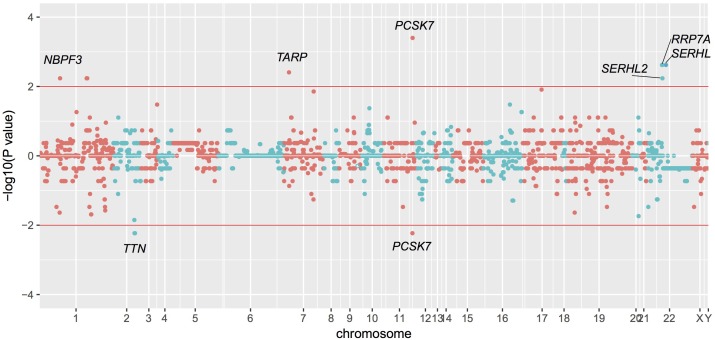
Manhattan plot of CNVs detected by whole exome sequencing. CNVs detection by statistical comparisons between the case and control groups. Different colors of points mean CNVs from different chromosome. *P* > 0 means duplication while *P* < 0 means deletion.

Based on these data, we identified statistically significant CNVs at different genomic loci. CNVs were filtered as potentially pathogenic if the *P* < 0.01 (Table 2). Finally, we identified eight potentially pathogenic CNVs of seven genes (*PCSK7, RRP7A, SERHL, TARP, TTN, SERHL2, and NBPF3*) among 45 patients with TAPVC (Figure 2). The percentage of subjects with CNVs candidates was 58.4% (45 of 78 TAPVC subjects).

**Table 2:** Copy number variant (CNV) detected by Fisher’ test.

CNV	Chromosome	Gene	Case with CNV	*P*-value
Duplication	Chr1	PCSK7	9	0.00040
Duplication	Chr22	RRP7A	7	0.00241
Duplication	Chr22	SERHL	7	0.00241
Duplication	Chr7	TARP	13	0.00394
Deletion	Chr2	TTN	8	0.00584
Duplication	Chr22	SERHL2	6	0.00584
Duplication	Chr1	NBPF3	6	0.00584
Deletion	Chr1	PCSK7	6	0.00584
Duplication	Chr17	KRTAP9-6	7	0.01239
Deletion	Chr2	TTN-AS1	5	0.01404
Duplication	Chr7	MTRNR2L6	5	0.01404
Deletion	Chr20	FER1L4	8	0.01818
Deletion	Chr1	FLG2	15	0.02036
Deletion	Chr1	FLG-AS1	15	0.02036
Deletion	Chr19	ZNF844	9	0.02292
Deletion	Chr1	CROCC	6	0.02292
Deletion	Chr1	OR2L5	6	0.02643
Deletion	Chr1	SPATA21	4	0.03347
Deletion	Chr1	RPTN	4	0.03347
Deletion	Chr11	AHNAK	10	0.03347
Deletion	ChrX	DMD	4	0.03347
Duplication	Chr3	MUC4	4	0.03347
Duplication	Chr16	STX4	4	0.03347
Deletion	Chr22	GGT3P	4	0.03347
Deletion	Chr1	OR2L3	4	0.03347
Deletion	Chr19	ZNF611	4	0.03347
Duplication	Chr10	ANTXRL	8	0.04273

### Expression Pattern in Human Embryonic Heart

We then detected the time course expression patterns of the candidate genes during different Carnegie stages of human heart development using microarray (Table 3). Expression of *TNN* in human embryonic hearts had a significantly higher level than other genes. Expression of *PCSK7*, *RRP7A*, and *NBPF3* were also high just behind *TNN.*

**Table 3:** Expression patterns of candidate genes in human embryonic hearts at different time points.

Chromosome	Gene	S10	S11	S12	S13	S14	S15	S16
chr12	GAPDH	17.47	17.50	17.48	17.45	17.35	17.24	17.00
chr11	PCSK7	7.01	7.04	7.05	7.07	7.18	7.17	7.05
chr22	RRP7A	7.00	6.93	7.03	6.69	7.05	6.93	6.88
chr22	SERHL	4.96	5.02	4.87	4.93	4.85	5.02	4.93
chr7	TARP	3.76	3.69	3.58	3.69	3.69	3.69	3.69
chr2	TTN	15.74	17.84	18.00	17.83	17.82	17.85	17.77
chr22	SERHL2	5.09	5.10	5.07	5.10	5.10	5.10	5.10
chr1	NBPF3	7.12	7.11	7.14	7.08	7.14	7.16	7.14

### STRING Network Analysis

**FIGURE 3:**
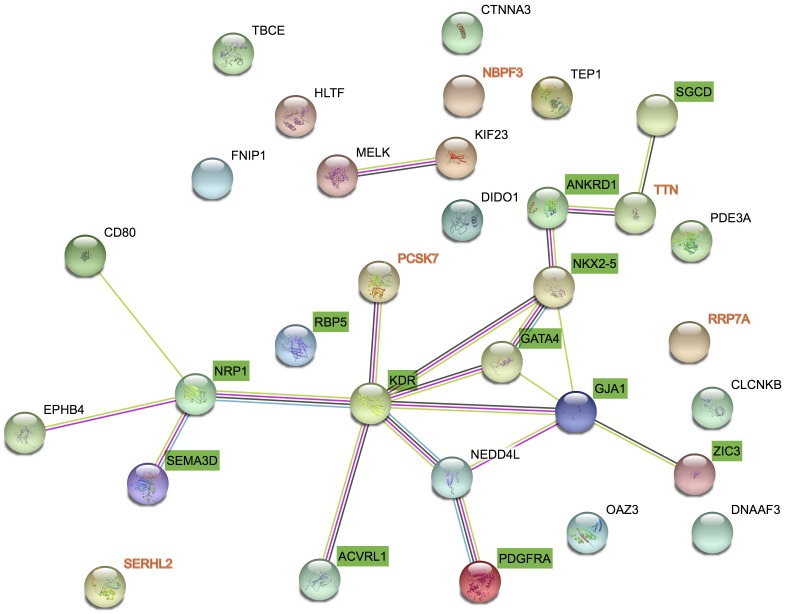
Protein–protein interaction between candidate genes and known genes. STRING networks included 27 genes previously associated to TAPVC and 7 candidate genes highlighted by CNV detection analysis.

We got 27 known pathogenic genes derived from the literature and publicly database. Then we used STRING database to explore the PPI network between CNV candidate genes and known pathogenic genes. Through PPI network, we found *PCSK7* and *TTN* had more direct and obvious relation to known pathogenic genes (Figure 3). *PCSK7* directly interacts with *KDR* and *TTN* indirectly interacts with *ANKRD1* and *SGCD*. These two genes could interact with other pathogenic genes via several other genes.

## Discussion

Total anomalous pulmonary venous connection is a rare congenital heart defect characterized by the misconnection of all four pulmonary veins, which could cause severe morbidity and mortality (Bando et al., 1996). Several genes have been associated TAPVC but the etiology of TAPVC is still complicated. To detect the underlying mechanism of TAPVC, we screened a cohort of 78 TAPVC cases and 100 healthy controls for rare CNVs and novel candidate genes, using whole exome sequencing (WES). Then we got seven totally novel candidate genes (*PCSK7, RRP7A, SERHL, TARP, TTN, SERHL2, and NBPF3*) that were associated with TAPVC. STRING network analysis demonstrated that *PCSK7* and *TTN* which are highly related to known pathogenic genes, appear to play an important role in the genetic mechanism of TAPVC.

Both deletion and duplication of CNV could have been associated with congenital disorders (McLysaght et al., 2014). Recent data show that the frequency of duplications is approximately half of deletions and their phenotypes of heart malformation are much more diverse. It is possible that genomic deletions are more likely to cause dosage sensitivity compared with duplications because the fold change is greater for deletions. In seven candidate genes, we found deletion CNVs only in *PCSK7* and *TTN.*

A total of 9 (9/78, 11.5%) patients had duplication and 6 (6/78, 7.7%) patients had deletion in *PCSK7*. *PCSK7* (Proprotein convertase subtilisin/kexin type 7) is a member of the subtilisin-like proprotein convertase family (Constam et al., 1996). The genetic regulation of PCSK expression especially *PCSK7* could bind to other genes to make huge impact on the blood pressure (Peloso et al., 2014; Turpeinen et al., 2015). Recent research of cardiovascular Disease (CVD) network using 1512 SNPs associated with 21 traits in genome-wide association showed *PCSK7* connected closely to the incidence of CVD (Yao et al., 2015). In our study, 8 (8/78, 10.3%) patients were detected to have the deletion in *TTN.TTN* (Titin) encodes the sarcomere protein titin. Among its related pathways are dilated cardiomyopathy (DCM) and cardiac conduction (Hinson et al., 2015). A large literature suggested that majority of familial and sporadic DCM had the rare variants in *TTN* (Herman et al., 2012; Ware et al., 2016). A study found *TTN* and *ANKRD1* which was an important pathogenic gene of TAPVC could combine to cause DCM (Arimura et al., 2009). And expression of *TNN* and *PCSK7* were higher than other genes in human embryonic hearts. Above all, *PCSK7* and *TTN* can be a totally novel candidate gene for TAPVC pathogenesis but the underlying mechanism remains unclear.

We found seven patients had duplications in *SERHL*, *RRP7A* and six patients in *SERHL2. SERHL*, *SERHL2*, and *RRP7A*, these genes are all located on chromosome 22q13. *SERHL* (Serine hydrolase-like) was encoded within the mRNA is an open reading frame of 311 amino acids which shows identity to a family of serine hydrolases (Sadusky et al., 2001). *SERHL* was found in tetralogy of Fallot patients and was associated with DNA methylation abnormalities (Serra-Juhe et al., 2015). *SERHL2* also belongs to the serine hydrolase family, while its functional role is yet to be elucidated, and other nearby genes in the region, such as *RRP7A*, could also be biological candidates linked to 22q13 deletion syndrome (Okada et al., 2018). Patients with 22q13 duplication have been reported to have the clinical diagnosis of cardiovascular abnormalities and intrauterine growth restriction (Chen et al., 2003; Rahikkala et al., 2013). The relationship between *RRP7A, SERHL*, and *SERHL2* and TAPVC needs to be further validated experimentally. Thus far, the functions of these genes in cardiovascular development remain unknown, and they be might newly associated with TAPVC pathogenesis.

In our research, *TARP* had the most patients than other genes, 13 (13/78, 16.7%) patients with duplication was detected in *TARP.TARP* (TCR gamma alternate reading frame protein) which is a marker for T cells and NKT cells and uniquely expressed in males in prostate epithelial cells and prostate cancer cells (Littman et al., 1987). It has been reported to be a biomarker for viral myocarditis (Rowe et al., 2018). We also found 6 (6/78, 7.7%) patients had duplication in *NBPF3.NBPF3* (NBPF member 3) is a member of the neuroblastoma breakpoint family (NBPF) which consists of dozens of recently duplicated genes primarily located in segmental duplications on human chromosome 1 (Vandepoele et al., 2005). NBPF3 is reported to express in a variety of tissues (Vandepoele and van Roy, 2007). Our study is flawed. First, the lack of parental samples limited our ability to study the genetic backgrounds of the variants. Second, we lack the information of prognosis of TAPVC cases. In addition, the functions of our candidate genes need to be further verified with fundamental research. In summary, an effective analytical bioinformatics strategy allowed us to identify CNVs in novel genes that play a vital role in TAPVC pathology. Based on the results of CNV discovery in a case-control cohort, our study found evidence that CNVs of seven candidate genes (*PCSK7, RRP7A, SERHL, TARP, TTN, SERHL2, and NBPF3*) could contribute to the genetic etiology of TAPVC. Our candidate genes open new fields of investigation into TAPVC pathology and provide novel insights into pulmonary vein development.

## Author Contributions

SC conceived and designed the project, responsible for the overall content and revised the manuscript. XS, LC, WL, JW, and XJ performed bioinformatics analysis of CNV data. BC, JS, YX, QF, and YaL collected the clinical data. ZL, GL, and YuL carried out all experiments. XS and LC prepared the manuscript. All authors have seen and approved the final manuscript.

## Conflict of Interest Statement

The authors declare that the research was conducted in the absence of any commercial or financial relationships that could be construed as a potential conflict of interest. The reviewer DZ declared a shared affiliation, with no collaboration, with several of the authors, XS, LC, XJ, BC, WL, JW, GL, YX, JS, QF, YaL, and SC, to the handling Editor at the time of review.

## Footnotes

1 http://dgv.tcag.ca/

2 http://omim.org

3 https://string-db.org/
